# The Mesencephalic Locomotor Region: Beyond Locomotor Control

**DOI:** 10.3389/fncir.2022.884785

**Published:** 2022-05-09

**Authors:** Brian R. Noga, Patrick J. Whelan

**Affiliations:** ^1^The Miami Project to Cure Paralysis, Department of Neurological Surgery, Miller School of Medicine, University of Miami, Miami, FL, United States; ^2^Hotchkiss Brain Institute, University of Calgary, Calgary, AB, Canada; ^3^Department of Comparative Biology and Experimental Medicine, University of Calgary, Calgary, AB, Canada

**Keywords:** locomotion, motor control, brainstem, spinal cord, dopamine, aminergic

## Abstract

The mesencephalic locomotor region (MLR) was discovered several decades ago in the cat. It was functionally defined based on the ability of low threshold electrical stimuli within a region comprising the cuneiform and pedunculopontine nucleus to evoke locomotion. Since then, similar regions have been found in diverse vertebrate species, including the lamprey, skate, rodent, pig, monkey, and human. The MLR, while often viewed under the lens of locomotion, is involved in diverse processes involving the autonomic nervous system, respiratory system, and the state-dependent activation of motor systems. This review will discuss the pedunculopontine nucleus and cuneiform nucleus that comprises the MLR and examine their respective connectomes from both an anatomical and functional angle. From a functional perspective, the MLR primes the cardiovascular and respiratory systems before the locomotor activity occurs. Inputs from a variety of higher structures, and direct outputs to the monoaminergic nuclei, allow the MLR to be able to respond appropriately to state-dependent locomotion. These state-dependent effects are roughly divided into escape and exploratory behavior, and the MLR also can reinforce the selection of these locomotor behaviors through projections to adjacent structures such as the periaqueductal gray or to limbic and cortical regions. Findings from the rat, mouse, pig, and cat will be discussed to highlight similarities and differences among diverse species.

## Introduction

Building on work by Graham-Brown, a renaissance in the study of locomotion started in the 1960s, driven by work by Anders Lundberg, Mark Shik, Grigori Orlovsky, and Sten Grillner (Stuart and Hultborn, 2008; Sharples and Whelan, 2020). It was generally recognized that the brainstem could elicit locomotor activity, coordinating in some way with spinal cord centers. This led to the publishing of work by Shik and Orlovskii in 1966 of an area of the brain bounded by the cuneiform (CnF) nucleus named the mesencephalic locomotor region or MLR, having a linear dimension of 1 mm, and when stimulated, produced locomotor activity (Shik et al., 1966, 1969). Anatomically the other nucleus that constitutes the MLR is the pedunculopontine nucleus (PPN). It appears that many principles are conserved across vertebrate species from lamprey to humans (Eidelberg et al., 1981; McClellan and Grillner, 1984; Garcia-Rill et al., 1985; Masdeu et al., 1994; Dubuc et al., 2008; Caggiano et al., 2018; Josset et al., 2018). While we use the term MLR in the review, it will be argued that it would be better to refer to the PPN and CnF separately, given the diversity of functions of the region.

## MLR Connectivity

**Figure 1:**
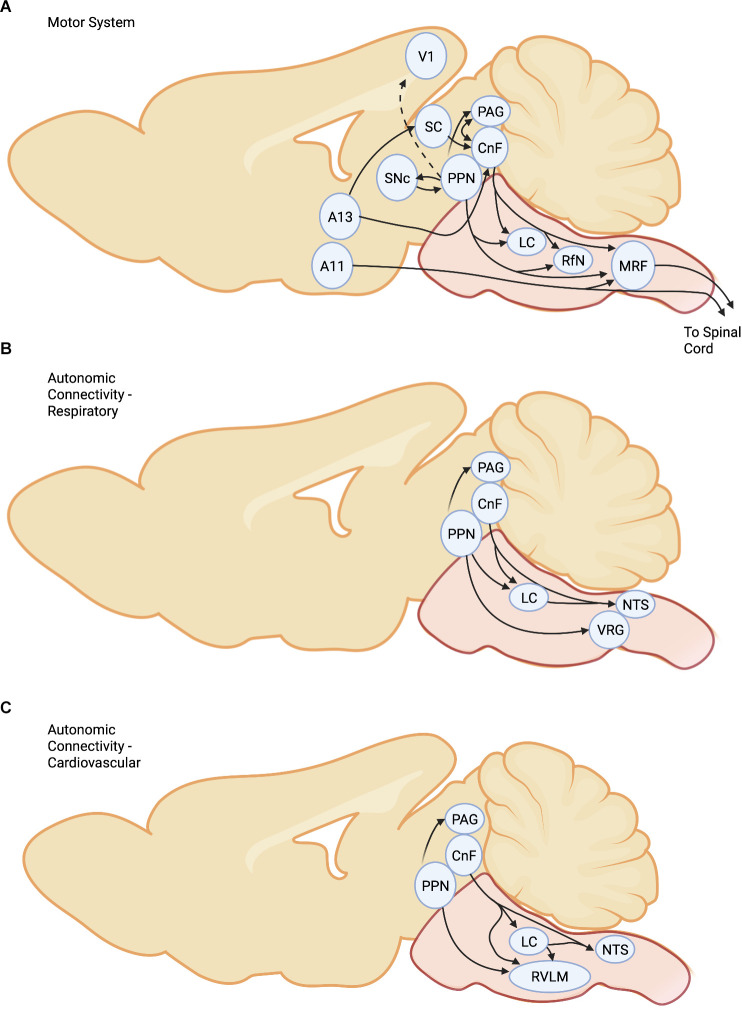
Connectivityof the MLR for **(A)** Motor System, **(B)** RespiratorySystem, and **(C)**Cardiovascular system. Abbreviations. *CnF*, CuneiformNucleus; *LC*, Locus Coeruleus; *PPN*,Pedunculopontine Nucleus; *SC*, Superior Colliculus;*SNc*, Substantia Nigra compacta; *PAG*, PeriaqueductalGray; *RfN*, Raphe Nucleus; *MRF*, Medullary ReticularFormation; *NTS*, nucleus tractus solitarii; *RVLM*,rostral ventrolateral medulla; *VRG*, Ventral Respiratory Group; *V1*, primary visual cortex.

The MLR forms a central node in the initiation of locomotion by higher brain centers (Figure 1A). It receives inputs from the ipsilateral subthalamic locomotor region (SLR; Orlovskii, 1969; Mel’nikova, 1977; Sinnamon and Stopford, 1987), the substantia nigra pars reticulata (SNr; Beresovskii and Bayev, 1988; Roseberry et al., 2016) and central amygdala (Roseberry et al., 2016). It is reciprocally connected with the contralateral MLR (Steeves and Jordan, 1984; Beresovskii and Bayev, 1988), possibly facilitating or coordinating descending signal output on both sides, and receives input from several sensory systems (e.g., auditory, visual) *via* the superior colliculus and lateral lemniscus, amongst others (Mitchell et al., 1988; Furigo et al., 2010; Roseberry et al., 2016). Activation of the MLR is also achieved by disinhibition of inhibitory SNr projections affecting both postural muscle tone and locomotion (Garcia-Rill et al., 1985; Takakusaki et al., 2003, 2016; Roseberry et al., 2016). Recent work suggests a greater normalized projection to the PPN compared to the CnF (McElvain et al., 2021). There is also a strong reciprocal interconnection with the periaqueductal gray (PAG; Mantyh, 1983; Beresovskii and Bayev, 1988; Sandner et al., 1992; Ferreira-Netto et al., 2005; Caggiano et al., 2018) which may be necessary for the mediation of rapid defensive decision making or the control of locomotion during the pursuit, initiated by activation of the amygdala (Han et al., 2017). It is important to consider the components of the MLR separately, as their functions are different. The differential effects of PPN stimulation on locomotion correspond to the diversity of anatomical projections to motor structures such as the cerebellum, spinal cord, basal ganglia, and brainstem. On the other hand, the more defined response of the CnF is consistent with projection patterns to downstream medial reticular formation (MRF) structures (Steeves and Jordan, 1984; Sotnichenko, 1985; Garcia-Rill and Skinner, 1987; Dautan et al., 2021). Similarly, projection patterns to the PPN are larger (basal ganglia and brainstem) than the CnF and more diverse (Caggiano et al., 2018; Dautan et al., 2021), and it has been suggested that the PPN is involved more in the modalities of movement rather than the execution of movement (Dautan et al., 2021). The output of the CnF is connected with PAG and other defensive areas of the brain suggesting an integrated escape functionality (Edwards and de Olmos, 1976; Steeves and Jordan, 1984; Dampney et al., 2013; Caggiano et al., 2018; Opris et al., 2019). The field is at an exciting juncture where electrophysiological data from pioneers such as Jankowska and colleagues examining supraspinal projections (Jankowska et al., 1993; Krutki et al., 2003) can now be married with circuit-specific modulation (Ferreira-Pinto et al., 2021) to establish sufficiency and necessity.

## MLR Function—A Tale of Two Nuclei

Considering both the CnF and PPN, the PPN has the most functional diversity. The PPN promotes arousal (Moruzzi and Magoun, 1949; Lee et al., 2014), likely through the ascending reticular activating system. Indeed, Parkinsonian patients implanted with deep brain stimulation (DBS) electrodes report side effects of increases in general arousal (Stefani et al., 2007). While chemogenetic stimulation of cholinergic neurons does not alter waking time, activation of PPN vesicular-glutamate transporter 2 (vGlut2) neurons has a robust effect on awake time (Kroeger et al., 2017). Moreover, in terms of locomotion, stimulation of the glutamatergic PPN cells induces locomotor activity, and these cells receive input from the amygdala and the basal ganglia (Roseberry et al., 2016, 2019). The results from stimulation of glutamatergic PPN cells on locomotion are mixed, with some suggesting they are involved in slow exploratory-like locomotor activity (Caggiano et al., 2018). Support for this notion is derived from increased time head-dipping in hole-board tasks, PPN unit activity during slower speeds, and optogenetic activity inducing slow locomotor activity (Caggiano et al., 2018). However, other reports suggest the opposite—that PPN stimulation produces locomotor arrest (Josset et al., 2018; Dautan et al., 2021). Another group reported arrest and locomotor behaviors in rats following optogenetic stimulation of the PPN but with most animals producing an abrupt increase in locomotor activity (Carvalho et al., 2020). Interestingly, photostimulation consistently produced either arrest or locomotion, suggesting that the subregion of the PPN stimulated was important. A recent study in mice shows that PPN vGlut2^+^photostimulation reliably inhibits the distance traveled by mice (Dautan et al., 2021). The lack of consensus may be due to the known heterogeneity of the PPN, differences in the viral spread, and the target region of the PPN (e.g., dorsoventral positioning; Chang et al., 2020). Unit electrophysiological recordings from PPN reinforce this point, with units positively and negatively correlated with locomotor speed (Carvalho et al., 2020). Finally, a recent study using a combination of gCaMP6 recording and loss or gain of function experiments provides evidence for a PPN to spinal cord projection involved in rearing (Ferreira-Pinto et al., 2021). Notably, few cells within the PPN in this study were modulated during locomotor activity.

PPN photostimulation associated with locomotor activity can produce activity in the V1 neurons of the visual cortex (Lee et al., 2014), through the basal forebrain bundle. So it is clear that the MLR, as well as producing locomotion, can feedback to cortical centers. Recently, a glutamatergic population was identified that projects from the PPN to the substantia nigra pars compacta (SNc), and which is involved in forelimb such as grooming and handling of objects (Ferreira-Pinto et al., 2021). This is interesting considering the newly discovered SNc to MLR projection (Ryczko et al., 2016; Fougère et al., 2019), suggesting a possible feedback mechanism. Combined with other work establishing a role for complex forelimb movements within the lateral rostral medulla (Esposito et al., 2014; Ruder et al., 2021), it suggests the brainstem is an integral part of the coding of multiple types of movements before the resultant command is relayed to spinal cord structures.

While the role of the PPN in locomotion is under debate, there is broad consensus that the CnF glutamatergic cells can initiate locomotion and control speed (Caggiano et al., 2018; Josset et al., 2018; Dautan et al., 2021). Low levels of stimulation promote walking, and the stimulus can be tuned to elicit different gaits (walk, trot, gallop, and bound). Extracellular recording confirms the CnF spike activity is more correlated to higher speeds compared to the PPN (Caggiano et al., 2018). This suggests that although the CnF could be recruited during normal walking, at higher levels of stimulation the locomotor activity patterns observed resemble escape.

## What Happens at Slow Speeds Vs. Fast Speeds—What Do We Know?

Locomotion elicited by stimulation of the MLR generally falls into two categories. Stimulation of the PPN can elicit slow-walking movements, along with arrest, while stimulation of the CnF can elicit locomotion across a much greater range. This difference was first reported by Orlovsky and colleagues, as discussed, and was later associated with vGlut2 positive neurons in both nuclei (Roseberry et al., 2016; Caggiano et al., 2018). When we examine the firing properties of vGlut2 neurons, they display diversity from rapidly adapting to non-adapting, while CnF neurons are mainly fast adapting. This may be associated with the heterogeneity of behaviors produced by PPN stimulation (Ferreira-Pinto et al., 2021) and the more diverse inputs to and projections of PPN vs. CnF (Caggiano et al., 2018; Dautan et al., 2021). However, we are at the beginning of a long journey to examine speed and other metrics of locomotion as they relate to the behaving animal. The activity of the MLR is related to the behavioral state. Reciprocal connectivity with structures throughout the brain ensures that the control exerted by the MLR fits the requirements of the behavioral state in conjunction with afferent feedback, limb coordination, and postural control (Mori, 1987; Mori et al., 1989). The CnF forms part of the defense circuit in rodents and other species (Mitchell et al., 1988). Glutamate and GABAergic cells have higher firing rates during cortical arousal. The PPN has extensive connectivity with dopaminergic and thalamic areas. Therefore, the idea has been proposed that the PPN could be a comparator region comparing expected and real situations and causing upstream state changes through the cholinergic system. The non-cholinergic system may contribute a different role in executing these changes to the motor system.

## MLR-What Have We Been Stimulating?

As mentioned, the MLR comprises mainly the CnF and the PPN. Still, since, especially in early work, it was based on electrical stimulus thresholds, it has not always been clear what structures have been stimulated. Indeed, the proximity of the two nuclei and the fact that they share a common border have often made it difficult to narrow the stimulation to one or the other. While the debate over the function of PPN and CnF has been ongoing for several decades (Whelan, 1996), even with less advanced techniques, evidence was already pointing to the cuneiform as being important for locomotor control (Inglis and Winn, 1995; Jordan, 1998). But even with multiple approaches including electrical, chemical, and optogenetic stimulation, there is still much debate regarding functional roles within MLR nuclei. Electrical stimulation of the MLR in cats can produce different latencies for evoked movement a fact noted for optogenetic stimulation of the PPN compared to CnF (Caggiano et al., 2018). Notably, electrode position, frequency, and current delivered are critical factors across different terrestrial species, including the cat, rat, mouse, and pig (Orlovsky et al., 1966; Garcia-Rill and Skinner, 1987; Noga et al., 2003; Chang et al., 2021b).

Latency of the response to electrical stimulation can vary, and this can be a function of the state, type of stimulus. In the unanesthetized pig, for a well-placed electrode, latency to onset of locomotion with electrical stimulation varies depending upon the amount of current injected (Chang et al., 2021b). Stimulation well above electrical thresholds for any particular frequency will evoke locomotion quicker and produce faster locomotion than if you stimulate at threshold strengths (Noga et al., 2017a). Furthermore, faster locomotion at onset is observed at threshold strengths with increasing stimulation frequency, suggesting that optimal stimulation frequency to engage the full range of walking gaits is lower, rather than higher. Stimulation history may also contribute to these effects. While the state of awake behaving animals can affect MLR stimulation, decerebrate animals also show diminished effectiveness with electrical stimulation related to whether the MLR has been stimulated for a long time, with repeated phasic stimulation inducing an inhibitory effect (Noga et al., 2009, 2011; Opris et al., 2019). Both photo and electrical stimulation have been used to examine MLR function, but there are differences in the activation mechanism between electrical and photostimulation. While optogenetics is useful for directly activating cell types, it is often compared with electrical stimulation. But it is important to note that electrical stimulation can activate fibers much more easily than cell bodies. That depends on stimulation configuration (whether cathodal, anodal, monopolar, or bipolar), the type of electrode (sharp or cylindrical) and the fiber orientations relative to the electrode. Electrical stimulation may activate diverse fibers leading to a mixture of monoamines, fast neurotransmitters, and neuropeptides being released onto target neurons. So, effects will depend on the various neurotransmitters’ combinatorial actions. This is compared to opsins that have been inserted into the membrane of neurons and which are then photostimulated or inhibited (Boyden et al., 2005). One can modulate specific circuits or specific neuronal phenotypes with the right tools. ChR2, the standard excitatory opsin activated by blue light, can follow stimulation frequencies up to approximately 30 Hz due to the kinetics of the opsin channel. However, fast opening channels, that would drive higher frequencies require stronger light. Red-shifted opsins, such as Chrimson, balance the need for stronger light for fast opening channels with greater channel expression at the membrane (Mager et al., 2018). From reported values for CnF and PPN unit frequencies, units generally fire between 5 and 50 Hz for most behaviors (Simon et al., 2010; Caggiano et al., 2018; Goetz et al., 2019; Carvalho et al., 2020). For work using ChR2 opsins, even when higher photostimulation frequency ranges (30–50 Hz) are reported neurons may not follow faithfully with spikes. Thus, the high firing frequency range of PPN and CnF neurons has not been fully probed and awaits work with opsins such as Chrimson and Cheta that follow higher stimulation frequencies.

## PAG—Inputs for Defensive Behavior

While not traditionally considered part of the MLR the proximity and importance in defensive behaviors warrant a discussion of this area. In prey species, defensive behaviors comprise both a flight and a freezing response. The decision to evoke flight or freezing responses is dependent on a combination of visual, auditory, and somatosensory inputs, and depends on subcortical systems. In this context, the PAG is an important mediator of defensive behavior (Figure 1A) (Bandler, 1982; Bandler and Carrive, 1988), including freezing or flight in response to threat (Kim et al., 2017; Koutsikou et al., 2017). The PAG is a complex structure and has four distinct regions: these comprise the dorsomedial (dmPAG), dorsolateral (dlPAG), lateral (lPAG), and ventrolateral (vlPAG) subdivisions (Carrive, 1993; Linnman et al., 2012; Dampney et al., 2013). Freezing responses are induced by neurons of the vlPAG (Bandler and Depaulis, 1988; Depaulis et al., 1989, 1992), which are glutamatergic and generally under control by local GABAergic neurons. Freezing is induced by projections to the pontomedullary (magnocellularis) reticular formation (Tovote et al., 2016). With short reorienting freezing responses, flight responses are induced by activation of the glutamatergic neurons within the dlPAG or the lPAG (Deng et al., 2016; Tovote et al., 2016). This response is likely mediated by indirect activation of reticulospinal (RS) neurons *via* an intermediary pathway to the MLR (Ferreira-Netto et al., 2005; Dampney et al., 2013) although a direct pathway has also been described for the monkey (Mantyh, 1983). Interestingly, dl/lPAG glutamatergic neurons inhibit vlPAG glutamatergic neurons by activating vlPAG GABAergic neurons, thus inhibiting the freezing response (Tovote et al., 2016). Based upon these results, MLR stimulation most likely activates (Opris et al., 2019) glutamatergic neurons of the dl/lPAG and GABAergic neurons of the vlPAG, facilitating locomotor activity and inhibiting freezing responses, respectively. The lateral hypothalamus (LH) provides an important projection to PAG, and photostimulation of GABAergic LH neurons contributes to prey detection and capture. In contrast, glutamatergic LH projections contribute to defensive responses (Rossier et al., 2021). The anatomical and functional linkage between the CnF, PAG, and limbic system (Edwards and de Olmos, 1976; Mantyh, 1983; Steeves and Jordan, 1984; Sotnichenko, 1985; Meller and Dennis, 1986; Ferreira-Netto et al., 2005; Dampney et al., 2013; Koutsikou et al., 2017; Caggiano et al., 2018) points to the important role played by the MLR in the integration of complex motor behaviors related to defensive behavior. The PAG receives other inputs associated with locomotion, such as the core of the nucleus accumbens, associated with reward-based locomotion, and the amygdala (Gross and Canteras, 2012; Tovote et al., 2016), which while traditionally associated with defensive responses is also associated with approach behaviors. Indeed, the amygdala also projects to the MLR, suggesting parallel pathways to initiate escape. While the connectivity between PAG and CnF is known, further studies regarding the interaction between these regions are required. Furthermore, it is possible that the PAG *via* MLR can influence visual processing through direct and indirect connectivity to the visual cortex. This may be another route whereby PAG can affect brain state (Lee et al., 2014).

## Monoaminergic Modulation

### Dopamine

Dopamine modulation of motor pathways was thought to be primarily indirect through the nigrostriatal pathway that projects *via* the basal ganglia to cortical and to brainstem regions (Figure 1A). The basal ganglia canonical circuit is highly conserved across vertebrates from lamprey to primates (Robertson et al., 2014). The direct D_1_R and indirect D_2_R pathways modulate output from the SNr and the globus pallidus internal onto motor centers. The SNr has GABAergic projections to the MLR, consistent with the indirect projections of the dopaminergic system (Roseberry et al., 2016). Thus, activating the dopaminergic nigrostriatal pathways leads to a removal of inhibition to the MLR through the D_1_ mediated direct and the D_2_ mediated indirect basal ganglia pathways. However, work demonstrates that the SNc A9 dopamine region or its analogs projects directly to the MLR in rat, salamander, lamprey, and mouse (Ryczko et al., 2013, 2016; Roseberry et al., 2016; Caggiano et al., 2018). Stimulation of the SNc increases extracellular dopamine concentrations in the MLR, and these effects are attenuated with D_1_R antagonists and potentiated with amphetamine. Interestingly there is also an ascending projection from the PPN to the SNc (Futami et al., 1995; Charara et al., 1996; Martinez-Gonzalez et al., 2011), which has been postulated to be involved in arousal, but equally could form a recurrent excitatory feedback loop that reinforces ongoing behavior. In mice, activity patterns within the SNc precede and are associated with locomotor activity, indicating that the direct projection to the MLR may increase activity within locomotor-related brainstem neurons (da Silva et al., 2018; Fougère et al., 2019). What additional role would a direct SNc link to the MLR have? The nigrostriatal pathway is one of massive convergence and compared to the SNr there are 800 times more projection neurons onto the striatum than the SNr projection neurons (Zheng and Wilson, 2002; Dudman and Krakauer, 2016). The direct SNc projections could be important for movement initiation or to integrate subcortical inputs with more fidelity than when filtered through the basal ganglia. Interestingly, the descending dopaminergic cells appear to co-localize glutamate, which provides a mechanism for fast excitatory activation of the MLR from the basal ganglia.

The other newly discovered pathway is the A13, a small nucleus within the medial zona incerta that projects to the CnF and, to a lesser extent, the PPN (Sharma et al., 2018, 2019). The A13 also projects to the superior colliculus and appears to be part of a defensive behavior circuit (Bolton et al., 2015). In contrast to the SNc descending circuit, A13 neurons co-localize GABA (Venkataraman et al., 2021), suggesting a dual fast inhibitory and a modulatory dopaminergic control of MLR function. The A13 also projects to the superior colliculus where there are D_1_ receptors located predominantly on GABAergic superficial neurons while D_2_ receptors are located in the deep layers (Bolton et al., 2015). This suggests both modulations of visual input and motor responses by the A13 within the superior colliculus (SC), which has been suggested to contribute to the salience of a visual object (Woolrych et al., 2021). An additional dopaminergic circuit can potentially modulate the motor function and is contiguous with the A13, but the A11 cell somas are noticeably larger and multipolar (Sharma et al., 2018). This is the A11 nucleus, contained within the posterior hypothalamus, which projects to all segments of the spinal cord (Björklund and Skagerberg, 1979; Skagerberg et al., 1982; Qu et al., 2006; Koblinger et al., 2014). Directly applied exogenous dopamine increases the excitability of lumbar motoneurons and interneurons and can potentiate locomotor activity (Humphreys and Whelan, 2012; Sharples et al., 2015, 2020). Optogenetic stimulation of the A11 led to an increase in bouts of locomotor activity suggesting a possible motor function for the A11 descending projection (Koblinger et al., 2018). The A11 and its descending spinal projections are discussed in more detail in other reviews (Sharples et al., 2014). In brief, it appears to be the sole source of spinal dopamine in rodents. Spinally applied dopamine can evoke diverse rhythms within the spinal cord, including episodic and locomotor activity (Humphreys and Whelan, 2012; Sharples et al., 2015, 2020, 2022).

There are other dopaminergic areas of importance for motor function and there is evidence that the PPN projections can alter the firing patterns of dopamine neurons, changing patterns from burst to tonic firing. The rostral PPN cholinergic and non-cholinergic neurons project widely to the dorsal striatum and can affect dopamine presynaptic release along with striatal neuronal firing (Dautan et al., 2014). Along with the direct dorsal striatal connections, indirect PPN connections to the dorsal striatum *via* the thalamus and ventral tegmental area (VTA) have been reported. Glutamatergic PPN neurons appear to project preferentially to the striatum compared to the CnF (Dautan et al., 2021). Neurons within the VTA show an increase in activity-dependent cFos activity during fictive locomotion produced by stimulation of the CnF (Opris et al., 2019). The VTA contains dopaminergic neurons involved in goal-directed behavior and reinforcement learning (Wise, 2004). It receives direct input from non-catecholaminergic neurons of the vlPAG (Suckow et al., 2013) and from cholinergic and glutamatergic neurons of the PPT and laterodorsal tegmental nucleus (LDT; Mena-Segovia and Bolam, 2017). Stimulation of cholinergic PPT terminals within the VTA activates dopaminergic neurons and transiently increases locomotor activity (Dautan et al., 2016). In contrast, LDT cholinergic neuron activation decreases locomotion (Dautan et al., 2016) and results in reward reinforcement (Xiao et al., 2016). These differential effects are likely due to actions on different neurons within the VTA. PPT glutamatergic neurons also increase arousal and drive motivated behavior *via* ascending projections, in part to the VTA (Kroeger et al., 2017; Yoo et al., 2017).

### Noradrenaline and Serotonin

The first demonstration of a key role for monoamines in activating spinal locomotor networks was the observation that intravenous noradrenaline and serotonin precursors produced reflex discharges in the spinal cat or rabbit that resembled locomotion (Jankowska et al., 1967; Viala and Buser, 1969). Soon after that, based on the resemblance of MLR evoked locomotion to the activity seen following l-3,4-dihydroxyphenylalanine (L-DOPA), Grillner and Shik (1973) postulated that the MLR activated a descending noradrenergic pathway controlling the spinal locomotor network. This idea was supported by the demonstration of noradrenergic neurons near the MLR, and that descending MLR projections included noradrenergic and serotonergic nuclei (Jordan and Steeves, 1976; Steeves and Jordan, 1984; Sotnichenko, 1985). Since then, multiple studies have shown that the spinal application of monoamines can initiate and modulate ongoing locomotor activity (e.g., Barbeau and Rossignol, 1991; Chau et al., 1998; Brustein and Rossignol, 1999; Musienko et al., 2011; Perrier and Cotel, 2015; Sharples et al., 2015, 2020). In spinal cord injured patients, a marked improvement in locomotor function and marked reductions in stretch reflexes and clonus may be obtained following oral administration of the noradrenergic alpha-agonist clonidine (Fung et al., 1990; Stewart et al., 1991). The neuromodulatory potential of monoamines varies by species. For example, in chronic spinal cats, noradrenergic agonists are most effective for enabling the initiation of locomotion (Barbeau and Rossignol, 1991; Marcoux and Rossignol, 2000) whereas, in the spinal rat and *in vitro* neonatal rat preparation, spinal application of serotonin or dopamine, with or without the co-application of N-methyl-D, L-aspartate, is most effective in eliciting stepping (Atsuta et al., 1991; Cowley and Schmidt, 1997; Sharples et al., 2015). In the cat, locomotor-activated neurons are innervated by monoaminergic fibers and express the serotonergic and noradrenergic postsynaptic receptors that mediate such effects (Noga et al., 2009, 2011). Spinal monoaminergic receptors are also found presynaptically on primary afferent and central terminals (Stone et al., 1998; Riedl et al., 2009), acting either as autoreceptors or heteroreceptors regulating transmitter release (Umeda et al., 1997; Li et al., 2000). Manipulation of endogenously released serotonin was shown to modulate the locomotor network in the *in vitro* neonatal mouse (Dunbar et al., 2010). That and the demonstration that cerulear and raphe neurons are activated during voluntary locomotion (Rasmussen et al., 1986; Jacobs and Fornal, 1999) suggested that monoaminergic modulatory pathways are engaged during locomotion, even though their activation is not obligatory for MLR evoked locomotion (Steeves et al., 1980). Subsequently, MLR stimulation was shown to activate noradrenergic and serotonergic nuclei of the brainstem (Opris et al., 2019) and result in the widespread release of noradrenaline and serotonin within the spinal cord during evoked locomotion (Noga et al., 2017b). Thus, descending monoaminergic pathways are activated in parallel with reticulospinal pathways during MLR-evoked locomotion and must be considered as a component of the descending locomotor pathway (Figure 1A).

## Visual System—Integration for Escape

The MLR can influence primary visual cortex V1 activity, *via* the basal forebrain, leading to increased gamma and reduced low-frequency oscillations (Lee et al., 2014). The other pathway is through the SC, which consists of an outer layer associated with vision and a deeper zone associated with motor and other functions (May, 2006). The SC is critical for triggering appropriate locomotor behavior. For example, when an approaching stimulus is presented to the upper visual field it evokes escape-like behavior (Yilmaz and Meister, 2013); in contrast, when approaching stimuli are displayed to the lower visual field, exploratory movements are evoked (Comoli et al., 2012). This is associated with predatory and appetitive stimuli, respectively. The upper visual field maps onto the medial SC, while the lower visual field projects onto the lateral SC. The medial SC projects onto the ipsilateral CnF with a smaller projection onto the contralateral PPN (Figure 1A) (Dean et al., 1989). Stimulation of the medial SC evokes locomotor movements similar to that evoked by the MLR, although the response is mediated by a projection onto GABAergic cells (Roseberry et al., 2016). It is not yet known what the transmitter types are that project from the SC to the MLR. Thus, either an excitatory or inhibitory projection onto CnF neurons is technically feasible. On the other hand, stimulation of the lateral SC produces contralateral orientating types of movements followed by exploratory movements (Sahibzada et al., 1986). The medial and lateral SC also project to the MRF. In contrast to its effects on V1 neurons, locomotor activity does not have a major effect on superficial SC neural activity, suggesting that the SC responds more faithfully to visual stimuli during movement (Savier et al., 2019).

## Autonomic Nervous System—Priming The System?

The MLR is intimately connected with the autonomic nervous system (Figures 1B,C). The link between the cuneiform and hemodynamic function was noted by Sirota and Shik (Sirota et al., 1970; Shik and Orlovsky, 1976) and has been confirmed since that period. Projections from the CnF project to areas associated with a cardiovascular function such as the rostral ventrolateral medulla (RVLM), PAG, locus coeruleus (LC), nucleus tractus solitarii (NTS), lateral paragigantocellular nucleus (LPGi) and parabrachial nucleus (Korte et al., 1992; Verberne, 1995; Lam et al., 1997; Shafei et al., 2012; Dampney et al., 2013; Netzer and Sévoz-Couche, 2021). Electrical stimulation of the CnF produces pressor responses in animals immobilized with neuromuscular blockers during fictive locomotion, activating neurons within several nuclei regulating blood pressure (Opris et al., 2019) and therefore is centrally coupled to the sympathetic system. Efferent connectivity from the CnF to the parabrachial and Kölliker-Fuse (KF) nucleus appears to mediate the sympathetic arm of the CnF (Korte et al., 1992). The PPN also contributes to the control of cardiovascular function likely through projections to the RVLM (Yasui et al., 1990). Interestingly, acetylcholine counteracts the pressor effect of CnF stimulation (Shafei et al., 2013) although elevations in sympathetic nerve activity, blood pressure, and baroreflex have been noted with chemical stimulation of the PPN in anesthetized rats (Padley et al., 2007). DBS stimulation of the PPN in Parkinson’s patients produces an elevation in blood pressure and baroreflex sensitivity (Hyam et al., 2019), which was particularly evident when the caudal PPN was targeted. On the other hand, CnF projections to the motor nucleus of the vagus and the NTS could be part of a proposed parasympathetic arm. Also, several nuclei are known to produce hypotension (Dampney and Horiuchi, 2003) and could be activated to counteract the pressor effect of MLR stimulation. Such nuclei include the nucleus ambiguous (Machado and Brody, 1988, 1990) and the dorsal motor nucleus of the vagus, possibly *via* a direct projection from the RVLM (DePuy et al., 2013). As part of a coordinated autonomic response, MLR stimulation results in cFos activation in the NTS (dorsal respiratory group), along with the retrofacial and lateral reticular nuclei (LRN—ventral respiratory group; Opris et al., 2019). Other nuclei associated with respiratory function include the raphe/parapyramidal region, LC/subcoeruleus, KF, PPT, and PAG (Kubin and Fenik, 2004; Gargaglioni et al., 2010; Dutschmann and Dick, 2012; Dampney et al., 2013; Subramanian and Holstege, 2014; Opris et al., 2019). Combined with previous work showing that stimulation of the hypothalamic and MLR facilitate respiratory rhythms and respiratory output (Sirota et al., 1971; Eldridge et al., 1981; DiMarco et al., 1983; Millhorn et al., 1987; Kawahara et al., 1989; Ezure and Tanaka, 1997), this points to an important role for the MLR in controlling respiratory function. Interestingly, respiratory activity increases before locomotor onset (Eldridge et al., 1981) indicating the preparatory nature of this control. Furthermore, treadmill exercise also activates neurons in many of the same areas (Iwamoto et al., 1996). Neurons within the LRN receive input from central respiratory and locomotor rhythms (Ezure and Tanaka, 1997) and are thought to transmit information of linked motor components to the cerebellum for eventual modulation of motor behaviors (Alstermark and Ekerot, 2013). In addition to cardiovascular and respiratory control, the PPN is reported to contribute to renal sympathetic nerve activity (Fink et al., 2017), bladder (Aviles-Olmos et al., 2011; Roy et al., 2018), In summary, we need to consider the MLR as part of a central controlling system that initiates locomotor and motor functions while concomitantly activating appropriate arms of the sympathetic and parasympathetic nervous systems. Furthermore, links with the autonomic nervous system coupled with the locomotor systems make the MLR an important target for coordinated recovery of multiple spinal cord centers following spinal cord injury. Notably, the pig, a valuable model for spinal cord injury research, also increases heart rate following MLR stimulation (Chang et al., 2021a). More research is required that carefully examines links with the autonomic nervous system using modern circuit-specific approaches and closed-loop feedback control (Noga and Guest, 2021).

## Understanding How The MLR Is Integrated from A Comparative and Translational Perspective

The MLR and specifically the PPN have been the focus of DBS trials designed to address movement disorders in patients. Primarily these patients have gait dysfunction (freezing-of-gait or FOG) because of Parkinson’s disease (PD). Initial reports were promising following DBS of the PPN, with motor scores and Unified Parkinson’s Disease Rating Scale improvements of 57% and 53%, respectively (Plaha and Gill, 2005). However, subsequent studies have shown mixed results as summarized in a recent meta-analysis (Wang et al., 2017). One possible issue is the dorsal MLR encompassing the CnF is critical and small differences in targeting produce significant effects on performance (Thevathasan et al., 2018; Goetz et al., 2019). These results correspond to results in rodents discussed previously, where PPN stimulation produces mixed effects while CnF produces generally consistent locomotory results. That said the type of cells and location within the PPN matter. Recent work found that activating caudal glutamatergic PPN neurons was particularly effective in rescuing locomotor activity (Masini and Kiehn, 2022). This rescue was independent of CnF glutamatergic neurons. Interestingly, activation of GABAergic PPN neurons effectively restored slow locomotor activity, which may suggest a combinatorial strategy in targeting neuronal populations within the MLR (Masini and Kiehn, 2022). More recently, the CnF has been promoted as an alternative target for FOG (Chang et al., 2020, 2021a) and a recent study in a mouse model of PD has shown that glutamatergic CnF neuron stimulation improves the initiation of locomotion while reducing the time spent immobile (Fougère et al., 2021). Preliminary results targeting the CnF in a PD patient with levodopa-resistant FOG demonstrate the procedure’s safety and show significant improvements in many gait parameters during CnF DBS (Chang et al., 2021a). Stimulation of the anterior CnF also showed significant increases in step length and velocity over that seen with either sham-DBS or PPN DBS (2-month period of DBS). Still, no significant improvements in clinical outcomes were observed for either DBS condition in PD patients with severe gait and balance disorders (Bourilhon et al., 2022). In addition to Parkinson’s disease, clinical trials are underway to determine if DBS of the MLR can improve function in incomplete spinal cord injured individuals. This work was a product of rodent work showing that MLR stimulation in a model with 80% of the cord damaged produced walking and swimming movements (Bachmann et al., 2013). Significant improvements in gait (stepping, electromyogram amplitude, speed, interlimb coordination, and joint excursion) are also observed following spinal contusion injuries in the pig (Noga et al., 2020). A detailed analysis of the efficacy of the CnF vs. the PPN has not been completed, but work in 6-OHDA mice shows that the CnF also is effective in augmenting locomotion (Fougère et al., 2021). The long translational timeframe since Shik and Orlovsky’s initial discovery of the MLR may rest on the necessity of stimulating the CnF, rather than the PPN. Ironically, they pointed out that the CnF appeared to be a better target for initiating locomotion more than 50 years ago. The MLR or its analog is found in diverse species from lampreys, skates, rodents, pigs, monkeys, and humans, and many similarities have been observed. But there are limitations to the translation of findings. For Parkinson’s disease, for example, no animal model to date can recapitulate the chronic pathology observed in humans. Due to bipedalism, differences in locomotor and postural control will presumably affect MLR connectivity and function.

## Future Directions

As we move forward, it will be critical to evaluate the role of the MLR in downstream connectivity to motor centers such as the MRF and spinal cord, connectivity to hemodynamic areas within the brainstem and spinal cord, and finally connectivity to cortical and limbic structures. To accomplish this, we need to deploy tools such as multi-site fiber photometry to record from these diverse areas. A limitation of both electrical and photostimulation is that the recruitment of populations tends to be synchronized and does not match the asynchronous firing of units observed. Overcoming this will likely take a combination of directed optogenetic activation of individual elements in cell populations coupled with closed-loop recordings (Shemesh et al., 2017). Another tool that is finally maturing are voltage sensors allowing all optical electrophysiology to be coupled with optogenetics. This has the potential of significantly moving the field forward since the spiking of populations of MLR neurons can be monitored to examine connectivity patterns (Fan et al., 2020). Cell-specific activation of the PPN and CnF will be critical in this endeavor as will be tagging activity with different behavioral states.

What is missing is the analysis of network connectivity, such as graph theory, to examine functional connectivity during the performance of different locomotor behaviors (Bassett and Sporns, 2017). An open question is whether the current behavioral tests provide a realistic portrayal of the diversity of behavioral states. To achieve this, we will need to develop more naturalistic testing environments. Finally, using diverse species to study MLR function is crucial (Chang et al., 2021b). This is critical not only for the inherent value of comparative biology but also for translational research leading to the development of new therapeutic approaches (Noga and Guest, 2021). This type of research is critical to explain the side effects of stimulation seen in the use of DBS, and indeed may necessitate the development and use of circuit-specific viral tools to ameliorate specific gait abnormalities.

## Conclusions

Our understanding of the MLR has evolved and while locomotion is one of the most reported outputs it has been clear for some time that it contributes to other functions. This is especially true of the PPN, where multiple motor behaviors have been reported such as rearing, grooming, and grasping. The PPN modulates other functions such as sleep-wake, arousal, and control of cardiovascular and respiratory function. On the other hand, the CnF while contributing to cardiovascular function, is more of a *bona fide* locomotor center. In line with PPN’s multiple roles, it shows a greater diversity of inputs than the CnF. In closing, the diversity of functions of the MLR should be kept in mind and it is our hope that this review encourages more collaborations with those from respiratory, cardiovascular, and motor neuroscientists.

## Author Contributions

Both BN and PW contributed to the writing and editing of the manuscript. All authors contributed to the article and approved the submitted version.

## Conflict of Interest

The authors declare that the research was conducted in the absence of any commercial or financial relationships that could be construed as a potential conflict of interest.

## Publisher’s Note

All claims expressed in this article are solely those of the authors and do not necessarily represent those of their affiliated organizations, or those of the publisher, the editors and the reviewers. Any product that may be evaluated in this article, or claim that may be made by its manufacturer, is not guaranteed or endorsed by the publisher.

## Abbreviations

CnF: cuneiform nucleus
DBS: deep brain stimulation
dlPAG: dorsolateral PAG
dmPAG: dorsomedial PAG
FOG: freezing-of-gait
KF: Kölliker-Fuse nucleus
l-DOPA: l-3,4-dihydroxyphenylalanine
LH: lateral hypothalamus
lPAG: lateral PAG
LC: locus ceruleus
LPGi: lateral paragigantocellular nucleus
LTD: laterodorsal tegmental nucleus
LRN: lateral reticular nucleus
MRF: medial reticular formation
MLR: mesencephalic locomotor region
NTS: nucleus tractus solitarii
PAG: periaqueductal gray
PD: Parkinson’s disease
PPN: pedunculopontine nucleus
RfN: raphe nucleus
RS: reticulospinal
RVLM: rostral ventrolateral medulla
SC: superior colliculus
SLR: subthalamic locomotor region
SNc: substantia nigra pars compacta
SNr: substantia nigra pars reticulata
vlPAG: ventrolateral PAG
vGlut2: vesicular-glutamate transporter 2
VRG: ventral respiratory group
VTA: ventral tegmental area
V1: primary visual cortex.
